# International fluoride symposium: effects of fluoride on human health and its mechanisms of action – a meeting report

**DOI:** 10.1186/s12919-025-00345-1

**Published:** 2025-09-29

**Authors:** E. Angeles Martinez-Mier, Gina A. Castiblanco-Rubio, Guillermo Tamayo-Cabeza, Andrea Aguiar, Morteza Bashash, Tracy Bastain, Kelly Brunst, Carrie Breton, Alejandra Cantoral, Jose L. Figueroa, Carly Goodman, Howard Hu, Jesus Ibarluzea-Maurolagoitia, Bruce Lanphear, Ashley Malin, Mauro Martinez, Karen E. Peterson, Elizabeth F. S. Roberts, Susan Schantz, Mikel Subiza-Perez, Marcela Tamayo-Ortiz, Martha M. Tellez-Rojo, Christine Till, Deborah J. Watkins, Frank Lippert

**Affiliations:** 1 School of Dentistry, Indiana University, Indianapolis, IN USA; 2 Department of Veterinary Biosciences, College of Veterinary Medicine, University of Illinois at Urbana-Champaign, Urbana, IL USA; 3 Department of Population and Public Health Sciences, Keck School of Medicine, University of Southern California, Los Angeles, CA USA; 4 Department of Environmental and Public Health Sciences, College of Medicine, University of Cincinnati, Cincinnati, OH USA; 5 Health Department, Universidad Iberoamericana, Mexico City, Mexico; 6 Center for Nutrition and Health Research, National Institute of Public Health, Cuernavaca, Morelos, Mexico; 7 Faculty of Health, York University, Toronto, ON Canada; 8 Department of Clinical and Health Psychology and Research Methods, University of the Basque Country UPV/EHU, Donostia-San Sebastián, Spain; 9 Faculty of Health Sciences, Simon Fraser University, Burnaby, BC Canada; 10 Department of Epidemiology, College of Public Health and Health Professions, University of Florida, Gainesville, FL USA; 11 Icahn School of Medicine at Mount Sinai, New York, NY USA; 12 School of Public Health, University of Michigan, Ann Arbor, MI USA; 13 Department of Anthropology, University of Michigan, Ann Arbor, MI USA; 14 Columbia University Mailman School of Public Health, New York, NY USA

**Keywords:** Fluorides*/toxicity, Dental Caries/prevention & control*, Child, Cognition, Humans, Prospective studies, Diet, Cohort studies

## Abstract

Fluoride prevents dental caries in a dose–response manner, leading some countries to adjust fluoride levels in water or table salt, as well as to promote the widespread use of topical fluoride. Recent studies have found associations between prenatal fluoride exposure levels of < 1.5 mg/L in water and urine and adverse neurodevelopmental outcomes. Although high fluoride levels have been recognized as neurotoxic in the past, a large body of contemporary evidence derived from retrospective analyses of birth cohort studies suggests fluoride may be neurotoxic to children at lower levels, highlighting the need for further, prospective research and multidisciplinary collaborations. The International Fluoride Symposium, held from April 29 to 30, 2024, brought together 20 researchers from the United States, Canada, Mexico, and Spain to discuss the impacts of fluoride on human health and its mechanisms of action. The primary goals of the symposium were to address challenges related to assessing fluoride exposure, share findings from cohort studies, develop a comprehensive research agenda, and foster international research partnerships. Key discussions included the dental caries preventive and toxic effects of fluoride, sources of fluoride exposure, biomarkers, dietary intake assessment methods, and analytical challenges. Presentation of results from cohort studies highlighted research on prenatal fluoride exposure and its association with neurodevelopmental outcomes and presented perspectives for future analyses. The symposium emphasized the need for customized dietary fluoride intake assessment tools, the development of high-throughput analytical methods for fluoride analysis, and research on the combined effects of fluoride with other chemical elements commonly found in the environment and the human diet. Additionally, there was a call for the harmonization of cohort data from diverse populations to address urgent questions about the impact of fluoride on human neurodevelopment and other health outcomes beyond oral health. It was agreed that prospective longitudinal cohort studies intentionally designed to assess fluoride exposure and neurodevelopment are essential, as none of the existing birth cohorts were designed to specifically study fluoride exposure (e.g., selection of biomarkers, collection intervals, diet exposure assessment). Furthermore, broader environmental health cohort studies that incorporate high-quality biomonitoring of waterborne neurotoxicants (such as fluoride, arsenic, lead, mercury), repeated measures of exposure, and inclusion of key covariates (e.g., socio-economic status, diet, iodine) are encouraged. Finally, developing effective communication strategies among scientists and the public was considered crucial for advancing fluoride research and mitigating potential health risks.

## Introduction

A robust body of evidence indicates that exposure to fluoride from various sources effectively helps prevent dental caries [1]. With that purpose, some countries have adjusted the fluoride levels in their community water supplies, while others have chosen to fluoridate table salt. Additionally, topical fluoride has become widespread for professional use (e.g., fluoride gels or varnishes) and at home care (e.g. toothpastes). However, excessive fluoride intake during tooth development can lead to dental fluorosis, an adverse health effect affecting enamel. Over the past three decades, dental fluorosis has become more prevalent in the USA, likely reflecting increased fluoride exposure and suggesting exposure from sources other than tap water and oral care products [2–4].

Recent studies have linked prenatal fluoride exposure to adverse neurodevelopmental effects, and research from birth cohort studies and animal models suggest that fluoride may act as a neurodevelopmental toxicant even at low levels of exposure [5]. Among the health outcomes studied so far in humans are reductions in Intelligence Quotient (IQ) and negative effects on behavior, anxiety, and depression. The World Health Organization (WHO) has set a safe limit for fluoride in drinking water of 1.5 mg/L. Although substantial evidence indicates that concentrations above the WHO limit can be neurotoxic, less data are available regarding effects at lower levels [5]. However, a recent meta-analysis of epidemiological studies investigating children’s IQ scores and prenatal or postnatal fluoride exposure found an inverse association between fluoride exposure and children’s IQ scores even at exposure levels currently considered safe [6]. This highlights the urgent need for a research agenda and the development of multidisciplinary collaborations to explore the effects of fluoride at exposure levels lower than the WHO limit, such as those commonly seen in community fluoridation programs.

### Symposium process

The International Fluoride Symposium took place at the Indiana University Indianapolis Campus Center over two days, on April 29th and 30th, 2024. The event brought together 20 researchers from the United States, Canada, Mexico, and Spain, all of whom have expertise in conducting studies investigating the effects of fluoride on human health and its mechanisms of action using data from cohort studies. The goals of the symposium were:

- To share identified challenges and limitations in fluoride exposure assessment and measurement.
- To share previous findings on cohort studies and share perspectives for the future
- To discuss and develop a research agenda.
- To provide a platform for establishing and strengthening international research partnerships.

On day 1, fluoride in the context of oral health and the methodological challenges associated with fluoride exposure assessment and measurement in biological samples were discussed, as well as past and future work developed by different cohort studies. On day 2, working groups were formed to discuss current methodological challenges and a research agenda to advance the science in this field. This report summarizes the presentations and discussions held by the participants.

## Fluoride in the context of oral health and methodological challenges of fluoride exposure assessment

### Fluoride’s preventive and toxic effects: an entangled web

#### Presented by E. Angeles Martinez-Mier

Dental caries (also known as tooth decay or cavities) is a multifactorial disease mediated by microbial biofilms that use sugar both as a fuel and a substrate to adhere to the dental structure and later produce acids that destroy that structure through a process called demineralization. Dental caries can significantly impact an individual's overall health and quality of life, and lead to reduced productivity [7]. The treatment of dental caries is costly and a burden for people living in both high and low-income countries [8]. While death directly caused by dental caries is rare, its presence can lead to years lived with disability [9].

Despite being a highly prevalent chronic disease, dental caries can be effectively prevented and controlled with fluoride [10]. Soon after the discovery of the caries preventive effects of fluoride, it was hypothesized that its mechanism was mainly “pre-eruptive”, meaning that fluoride ingested from fluoridated water or salt, for example, incorporates into the developing tooth structure to make it more resistant to acid attacks [11]. However, current evidence has rejected that hypothesis. We now know that teeth benefit from fluoride only after their appearance in the oral cavity (“post-eruptive” effects) and that fluoride aids in replacing the lost minerals through a process called re-mineralization [12]. Regular exposure to small amounts of fluoride within the oral environment, such as direct contact of the tooth structure with fluoridated water or toothpaste, can delay demineralization and enhance remineralization [13].

Pre-eruptive effects are sometimes called “systemic”, while post-eruptive effects are also called “topical”. However, these terms should not be used interchangeably: pre- and post-eruptive refer to the timing of fluoride exposure. In contrast, systemic and topical refer to the mode of administration or source of fluoride. Current evidence indicates that defining the effects of fluoride from a specific source solely as “topical” or “systemic” is not entirely accurate [14]. For example, although toothpaste is used topically, if the toothpaste is swallowed (which may occur among young children), the exposure occurs both topically and systemically. It is important to understand that regardless of the mode of administration, fluoride will only be effective if it is present in the mouth when caries are developing.

Systemic exposure or intake can be excessive and lead to a defect of the enamel structure called dental fluorosis [15]. An increase in the prevalence of enamel fluorosis has raised questions regarding a possible excessive intake of and exposure to fluoride in children in both fluoridated and non-fluoridated communities [3]. Since the discovery of the preventive effects of fluoride, food sources have diversified, as well as the sources of fluoride intake and exposure. In this context, it is imperative to make a contemporary assessment of fluoride exposure, its sources and contribution to total exposure. Unlike other environmental exposures without beneficial effects (e.g., lead), fluoride does have proven benefits to human health and remains the most cost-effective agent for preventing and controlling dental caries [16, 17].

### Sources of fluoride exposure, metabolism in the human body, and its relationship with biomarkers

#### Presented by Gina A. Castiblanco-Rubio

Ionic fluoride is widespread in the environment. It is commonly found in soil, industrial emissions, several pesticides, and leaches into agricultural products, which makes it a trace element in the human diet [18]. Fluoride has been added to water supplies or table salt in some countries to prevent and control dental caries at the community level [19, 20] and it is a component of various oral healthcare products. Therefore, a comprehensive assessment of fluoride exposure strives to capture as much variability as possible from different sources, ideally through biomarkers.

The choice of a biomarker should be guided by both the knowledge of the kinetics of fluoride in the human body, and the window of exposure being assessed. In humans, plasma constitutes the central compartment through which fluoride is distributed to soft tissues and deposited in hard tissues [21] and is considered a marker of recent exposure. Some fluoride will be deposited into hard and/or keratinized tissues, which grow at a slow pace and therefore represent longer exposure windows. The remaining fluoride (not deposited in hard and/or keratinized tissues) is eliminated through the urine within 24 h [22] Therefore, plasma [23], urine, nails [24], and teeth are biological media that have been used or are currently being studied as biomarkers of fluoride exposure. For detailed reviews on fluoride biomarkers, we recommend consulting Pessan & Buzalaf [25] and Rugg-Gunn et al. [26].

No single biomarker of fluoride exposure is suitable or comprehensive to address all research questions. Ideally, the exposure window represented by a biomarker should align as closely as possible with the timing of the development of the health outcome of interest. Dilution-adjusted spot urinary fluoride levels have gained popularity as a biomarker due to their practicality and have been validated against sources of exposure (e.g., water), but their limitations must be considered when interpreting the results [27]. Several physiological factors that affect the concentration of fluoride in different tissues or fluids must be considered, including (but not limited to) age, intra- and interindividual variability, acid-base balance, and genetics [21]. Research on biomarkers of exposure is continuously evolving, and it is advisable to consult with experts on fluoride exposure and metabolism to select the most appropriate biomarker for specific research questions.

### Dietary fluoride intake assessment methods: advantages and limitations

#### Presented by Alejandra Cantoral and Gina A. Castiblanco-Rubio

The assessment of dietary fluoride intake is complex due to its widespread presence in various foods, beverages, supplements, and dental products. Dietary assessment methods often underestimate fluoride intake due to reporting inaccuracies and challenges in quantifying fluoride in fluoridated water or salt. Duplicate diets, 24-h recalls, and food frequency questionnaires (FFQs) are among the methods used to estimate fluoride intake in epidemiology.

Duplicate diets are the gold standard dietary assessment of fluoride exposure from the diet (e.g., food, beverages) but they do not consider other sources of fluoride exposure [28]. In this method, participants serve themselves or are served meals as they normally would on a typical day, replicating the servings on a separate plate. After the meal, researchers collect the duplicated meal. Then, the sample is stored and later processed in the laboratory. This approach minimizes estimation errors, provides a direct representation of food intake, and allows for immediate observation of food choices. However, its high cost and time requirements make it impractical for large-scale studies [29].

The 24-h recall method ranks second for assessing dietary fluoride intake. Participants recall all foods and beverages consumed over the previous day, detailing types, cooking methods, quantities, and consumption times, and then dietary intake is estimated using fluoride concentration reported in dietary databases [30]. While this method is effective for short-term dietary assessment and allows for comparisons between groups or individuals, it is also reliant on memory, introducing potential recall bias. For example, large portion sizes are often underestimated whereas small portion sizes are generally overestimated in recall studies [31]. As it may not capture habitual dietary patterns, using multiple 24-h recalls (ideally, two non-consecutive weekdays and one weekend day) is recommended to improve accuracy. It is also important to mention that due to the rapid elimination of fluoride through the urine, urinary fluoride levels in 24-h collections are only correlated with dietary intake over the same period of time [32]. Other methods, combining above approaches have also been evaluated, such as 3-d food diaries and the 2-d duplicate plate method. Both have been shown to be comparable in their assessment of dietary fluoride exposure [33]. However, assessments over multiple days require greater compliance by study participants than the (1-d) duplicate plate and 24-h recall methods.

FFQs collect data on the frequency of different foods and beverages consumed over a specific period, typically a month or a year [30]. This method provides insights into habitual diets and is useful for studying long-term trends. It is easy to administer and cost-effective but has weaknesses, such as dependence on the participant’s memory, which can also lead to recall bias. FFQs typically overestimate food intake compared to other methods [34]. Additionally, the list of foods in FFQs is often limited, potentially omitting key items relevant to fluoride intake, such as bottled water, tap water, and salt. To enhance the FFQ's effectiveness in fluoride estimations, it is advisable to adapt the questionnaires to include relevant items and follow-up questions for those requiring extra information, such as tap water sources, brands of bottled water, and the amount of salt used for cooking and at the table, to name a few.

Once estimated, fluoride intake can be reported in two ways: as an absolute daily intake (mg F/d) or as an exposure dose per unit of weight (mg F/kg/d). Choosing between these methods depends on the goals of the analysis. Absolute values facilitate comparisons to dietary guidelines [35], while exposure levels are useful for risk assessments. However, using doses can pose challenges in epidemiological studies examining the relationship between fluoride intake and health outcomes, as dividing absolute intake by body weight might underestimate exposure, as fluoride is not distributed to fatty tissues. Furthermore, depending on the goals of the analysis, adjustment for weight and/or energy intake may be relevant to separate the effects of fluoride from those of higher or lower calorie intake [30]. When examining the effects of fluoride on health outcomes, it is recommended to include absolute values in statistical models along with relevant covariates such as weight and/or energy intake, depending on the case.

Other key variables to consider when using dietary fluoride intake in statistical models include, for example, calcium intake, iodine intake, and diet composition [21]. Calcium intake decreases the intestinal absorption of fluoride [36] and may modify the release of ions stored in the bone [37] (including fluoride). Also, due to their tendency to alkalinize urine, vegetarian diets can increase fluoride excretion [21]. Dietary indexes estimating acid load [38, 39] (as a proxy of diet composition) may also serve as useful covariates, though their relevance in epidemiological analyses needs further testing.

An appropriate understanding of the advantages and limitations of dietary assessment methods, within the context of fluoride intake and metabolism, can greatly improve estimations of dietary fluoride intake and its use in statistical models.

### Potentiometric analysis of fluoride: biomarkers, sample collection, and analytical challenges

#### Presented by Guillermo Tamayo-Cabeza and Frank Lippert

Depending on the timing of fluoride exposure intended to be estimated, biomarkers of fluoride can be categorized as either *contemporary* (markers of recent exposure) or *historical* (markers of past exposure). Fluoride concentrations in plasma, urine, and nails reflect contemporary fluoride exposure - each with a different, but recent timeframe; while fluoride concentrations in teeth are considered historical biomarkers, as fluoride accumulates in the mineral structure over several years.

- Fasting plasma fluoride levels from venous blood [22] reflect an individual’s “baseline” or usual fluoride levels (which are dependent on factors such as fluoride exchange with bone). If measured in samples collected during overnight fasting, plasma fluoride levels are not affected by fluctuations in dietary intake and are known to be moderately correlated with water fluoride levels in other body fluids such as saliva and amniotic fluid. However, collecting and analyzing these samples can be challenging due to higher costs, adherence to fasting protocols, the management of sample collection, transport and storage, and analysis in the laboratory. In particular, volumes up to 3 mL may be required for reliable analysis with potentiometric methods.
- Urine collected over 24 h reflects fluoride exposure over the past day and is strongly correlated with dietary intake in the same period of time [32]. The primary challenges in using this biomarker include costs, ensuring protocol compliance and addressing sex-related differences in collection practices. Attempts have been made to replace 24-h collections with their more convenient counterpart: dilution-adjusted spot urine samples. However, although more practical and convenient, spot urine samples are less reliable due to diurnal variations (some researchers use first void while others use voids at any time of the day), spikes due to dietary intake and individual factors [40]. Furthermore, different methods to adjust urinary dilution are used, such as urinary creatinine or specific gravity [41], with the latter being more adequate for the fluoride analyte. For dilution-adjusted urinary fluoride levels to be valuable indicators of fluoride exposure, it is still critical to standardize collection and analysis protocols.
- Nails are also contemporary biomarkers of fluoride exposure. Toenails are preferred over fingernails due to reduced external contamination risk and are promising novel biomarkers [25]. However, variability in individual growth rates and the unsuitability of some nails for analysis (such as being covered with nail polish), are challenges. The nail collection process involves removing polish, clipping nails after bathing, and pooling samples to obtain sufficient mass for analysis.
- Teeth, particularly exfoliated primary and extracted permanent teeth, are reliable indicators of historical fluoride exposure [25]. To obtain accurate measurements, enamel or dentin require careful preparation, including cleaning, ashing, and grinding. Challenges with this type of sample include the invasive nature of collection, the wait involved with the natural exfoliation of primary teeth, and the potential for dentin loss or contamination during processing.

After selecting the appropriate biomarker to answer a research question, choosing the proper method to measure fluoride concentrations is critical. Potentiometric methods (those using a fluoride-specific electrode) have proven to be reliable and accurate when the samples meet certain conditions. Most aqueous samples with volumes ≥ 1 mL can be analyzed directly with the fluoride ion-specific electrode, while non-aqueous samples and complex mixture samples require preparation to diffuse and concentrate fluoride for potentiometric analyses. Due to potential interferences, direct potentiometric methods (those not requiring sample preparation) are often unsuitable for fluoride analysis in complex biological matrices. Factors such as ionic strength, pH, and temperature must be carefully controlled to ensure accuracy. A widely used indirect method is aided by hexamethyldisiloxane (HMDS), a volatile compound that helps diffusing and concentrating fluoride ions [42]. However, the extraction efficiency of this method can significantly impact its accuracy, especially at low fluoride concentrations that fall below the limit of detection of the fluoride selective electrode (0.019 mg/L).

Lastly, the lack of standardized methods for fluoride determinations can contribute to variability in results across laboratories. Previous efforts aiming to develop and standardize ion-selective electrode-based methods tailored for fluoride analysis have demonstrated to improve accuracy and consistency [43]. Furthermore, potentiometric methods require experienced personnel and are unsuitable to handle many samples. Sensitive and reproducible high throughput methods are needed for the measurement of fluoride in biological samples.

### Fluoride may not be the only elephant in the room: assessing the impact of fluoride exposure within chemical mixtures on health outcomes

#### Presented by Gina A. Castiblanco-Rubio, Deborah Watkins, Marcela Tamayo-Ortiz, and Ashley Malin

Human populations are commonly exposed to inorganic fluoride as part of chemical mixtures rather than in isolation. At least six chemical elements commonly found in the environment and the human diet have the potential to interact with fluoride. These include calcium, iodine, lead, arsenic, mercury, and aluminum. Consuming fluoride with calcium-rich foods can decrease fluoride absorption [36]. Fluoride exposure may also exacerbate the adverse metabolic effects of insufficient calcium intake on calcified tissues [44]. Additionally, fluoride may intensify the impact of iodine deficiency on thyroid function and elevate lead concentrations in blood and calcified tissues, likely by indirectly competing with lead for storage in the bone structure [45]. Little is known, however, how fluoride can impact the effects of abundance of iodine. Other potentially significant interactions include those of arsenic and aluminum, the mechanisms of which are yet to be studied [46]. Mercury is often co-present in highly polluted areas [47], dental amalgams, and some fish [48].

Recent progress has been made in the study of simultaneous exposure to fluoride and metals. For example, a study involving a sample of pregnant individuals in Los Angeles, California, revealed associations between urinary fluoride levels and metals such as cadmium, lead, and barium [49]. Additionally, in a cohort of Canadian adults, moderate to severe iodine deficiency modified the potentially harmful association of fluoride exposure with thyroid function [50]. These findings emphasize the importance of considering multiple exposures when evaluating the impact of fluoride on human health.

Assessing the impact of fluoride on health outcomes in the context of chemical mixtures presents conceptual and statistical challenges. For example, individual chemicals within a mixture may be highly collinear, display complex interactions, or non-linear dose–response functions [51]. However, a range of statistical methods developed and advanced over the past decade allow us to evaluate the impact of chemical mixtures on human health more effectively. The selection of an appropriate method is guided by the research goal [52], which may include: 1) the identification of specific components of the mixture related to the health outcome; 2) understanding how the different components of the mixture interact to influence the health outcome; or 3) determining the overall effect of the mixture on the health outcome. However, results from mixtures analyses may be difficult to interpret for regulatory purposes, which is an important consideration as mixture methods continue to evolve [51, 52].

For health outcomes already associated with fluoride exposure, future research should consider assessing the effect of chemical mixtures containing fluoride while considering the biological plausibility of the proposed hypotheses. For instance, the compounds used for the fluoridation of public water supplies are complex chemical mixtures themselves [53], yet the way these chemicals interact to produce an overall effect is unknown. As statistical methods for analyzing environmental mixtures become more widely implemented and available, investigating an everyday exposure like fluoride in the context of mixtures could help to address the most pressing issues of fluoride research, environmental, and public health.

### Birth cohort studies: state of the evidence

In the early days of fluoride research, study designs were primarily experimental or cross-sectional to address specific questions related to fluoride and its impact on oral health outcomes. In parallel, environmental health research has developed methodologies to enhance the cost-effectiveness of studies on environmental exposures and their effects on human health. This includes the use of longitudinal designs and population-based maternal and birth cohort studies that allow for the assessment of multiple environmental exposures and health outcomes over time. These studies collect and store data from questionnaires, clinical examinations, and biological samples, enabling researchers to not only address the original questions but also to meet other research needs that may emerge in the future. A National Toxicology Program (NTP) Monograph, published in August 2024, systematically evaluated over 70 observational studies and concluded—with moderate confidence—that higher fluoride exposure is associated with lower IQ in children [5]. A subsequent meta-analysis supported this finding, reported a statistically significant inverse relationship between fluoride exposure and children's IQ scores [6]. Notably, among the best available evidence (i.e., the high-quality studies), inverse associations were observed even when fluoride exposure was restricted to less than 1.5 mg/L as estimated by measurements in both urine and drinking water. Three high-quality prospective birth cohort studies that took multiple urinary measurements throughout pregnancy were identified: two, conducted in regions where maternal fluoride exposure levels were comparable to those in the United States, reported significant inverse associations with children’s IQ [54, 55], the third, conducted in an area with lower maternal fluoride exposure, found no association with children’s IQ [56]. This has motivated the use of existing resources from cohorts in regions with lower fluoride, such as the Americas and Europe. In the International Fluoride Symposium, researchers with previous or ongoing work with maternal and birth cohorts in the USA, Canada, Mexico, and Spain presented their previous, ongoing and future work answering fluoride exposure-related questions.

### The early life exposures in Mexico to ENvironmental Toxicants “ELEMENT” and the programming research in obesity, GRowth, environment, and social stress “PROGRESS” studies

#### Presented by Martha M. Tellez-Rojo

The ELEMENT study, initiated in the mid-1990s, is a comprehensive, multi-institutional, and international research initiative tracking approximately 600 mother–child pairs from three birth cohorts in Mexico City for up to 30 years (Cohort 1 recruited between 1994–1995, Cohort 2 between 1997–2000, and Cohort 3 between 2001–2003). Since its inception, the project has systematically collected data from participants at various stages of life, including the prenatal period, early to mid-childhood, adolescence, and young adulthood. This extensive follow-up has provided invaluable insights into how environmental exposures to metals and chemicals during critical developmental periods, such as pregnancy and puberty, can influence maternal and child health outcomes. The research underscores the significance of early-life environmental factors in shaping long-term health trajectories.

The PROGRESS longitudinal prebirth cohort, established in 2007, aims to examine the interplay between social stressors, environmental factors, and molecular biomarkers throughout pregnancy and childhood. Over 17 years, PROGRESS has followed 650 mother–child pairs, collecting extensive data on environmental exposures during pregnancy and childhood. The study employs social science, epidemiology, and toxicology methodologies to understand transdisciplinary risk factors impacting health. A key strength of PROGRESS is its integration of multiple research domains to assess cumulative and interactive effects of exposures since the prenatal period. This approach helps identify critical periods of vulnerability and resilience, informing targeted interventions.

Over recent years, the ELEMENT and PROGRESS teams have explored the impact of fluoride exposure, particularly on neurodevelopment outcomes. In 2017 and 2018, we observed that higher maternal urinary fluoride during pregnancy was linked to lower cognitive performance, including lower Full-Scale IQ scores [57] and increased inattention behaviors in children [58]. Subsequent studies corroborated these findings in 2020 and 2022, where prenatal fluoride exposure in urine was linked to IQ decrements, and non-verbal abilities were more vulnerable [54]. Additionally, we found no significant correlation between sociodemographic factors and fluoride biomarkers during pregnancy [59]. Since table salt is fluoridated in Mexico, we pioneered the development of a database of fluoride content in foods in Mexico [60], and we found an association among dietary fluoride exposure during pregnancy, and lower cognitive outcomes in boys [61]. Furthermore, we found no significant association between concurrent daily food and beverage fluoride intake and children’s urinary fluoride, as collected in our studies. However, we identified that urinary fluoride levels in Mexican children are comparable to that in fluoridated Canadian communities [62] and a fluoridated community in Los Angeles, CA, USA [63]. We also found that as of 2021, estimated dietary fluoride intake levels in pregnant women in Mexico were below those recommended for caries prevention [64], while calcium supplementation reduced urinary fluoride levels in this subpopulation group [65].

Our work also extended to cardiometabolic and other health outcomes. In 2020 and 2024, we reported that higher plasma fluoride levels were associated with increased body fat, blood pressure, and cardiometabolic risk factors in peripubertal girls [66]. We also found a link between dietary fluoride and lipid profile and HbA1c levels in children [67]. Research in 2019 and 2021 revealed that urinary fluoride exposure during peripubescence was associated with later pubertal development in boys [68] and higher fluoride intake with lower rates of dental caries in adolescents [69]. Moreover, we identified that early pregnancy urinary fluoride levels were associated with increased birth weight and length [70]. However, no associations were found between low-level childhood fluoride exposure and renal function [71].

### Maternal-Infant Research on Environmental Chemicals (MIREC), Canada

#### Presented by Christine Till

The MIREC study is a pan-Canadian prospective pregnancy and birth cohort designed to determine whether maternal exposures to environmental chemicals are associated with adverse pregnancy and child health outcomes. Pregnant women (*n* = 2001) were recruited between 2008 and 2011 in their first trimester of pregnancy from ten cities: Vancouver, Edmonton, Winnipeg, Sudbury, Ottawa, Kingston, Toronto, Hamilton, Montreal, and Halifax. Information about the cohort profile, recruitment details, and follow-up are described elsewhere [72, 73].

Fluoride exposure has been measured in mother–child pairs using biomarkers (urine, teeth), water records, and questionnaire data assessing dietary fluoride intake. Over 5000 spot samples were collected serially over the course of pregnancy (Trimester 1, *n* = 1885; Trimester 2, *n* = 1738, and Trimester 3, *n* = 1660). Urinary fluoride levels were also measured in 654 children between the ages of 2–5 years. In addition, water fluoride data were collected from water treatment plants, with half adding fluoride, and linked to MIREC participants by matching their postal codes to treatment plant region and the year of pregnancy.

The MIREC study is to date, the world’s largest biomonitoring study of fluoride levels in pregnant women. Results showed that women living in fluoridated communities have approximately twice the level of urinary fluoride compared with women living in non-fluoridated communities [27], with drinking water and black tea identified as major sources.

Associations between fluoride exposure and child neurodevelopmental outcomes have been studied in the MIREC cohort. Key findings include an association between higher urinary fluoride concentrations in pregnancy and lower child IQ in males [55]. A stronger association with IQ was seen for prenatal (maternal) versus concurrent (childhood) fluoride exposure [74], and among mothers with low iodine status (< 200 μg/g) [50]. In addition, higher fluoride levels in tap water during the first 6 months of life were associated with lower Performance IQ in both formula and breastfed infants, even after controlling for prenatal fluoride exposure [75]. Increasing fluoride levels in drinking water were also associated with poorer visual acuity and a less mature autonomic nervous system in infants [76].

Recent work found that pregnant women who drank fluoridated tap water had reduced thyroid activity [77] and alterations to thyroid hormones [78]. Pregnant women who drank fluoridated tap water for more than one year were almost twice more likely to have clinical hypothyroidism. Those who did not have an autoimmune thyroid disorder were almost four times more likely to have hypothyroid disease.

Ongoing work includes measuring fluoride and other metals in tooth dentin using 550 deciduous teeth collected from 350 children in the MIREC cohort. Measures of dentin fluoride will be used to test whether cumulative fluoride exposure in tooth dentin is associated with lower IQ score and diminished attention. This future work will assess windows of vulnerability.

## New Hampshire Birth Cohort Study (NHBCS), New Hampshire, USA

### Presented by Carly Goodman and Guillermo Tamayo-Cabeza

The New Hampshire Birth Cohort Study (NHBCS) is an ongoing prospective birth cohort study which began in 2009. NHBCS is designed to examine how contaminants in drinking water and food affect the health of pregnant women and their children [79]. A concurrent study is examining fluoride exposure in 578 mother–child pairs from the NHBCS, an ongoing prospective study Child neurodevelopmental outcomes (IQ scores, behavioral ratings, motor outcomes), maternal toenail clippings and urine samples, water samples as well as detailed information about the frequency and quantity of water and food intake have been collected in 464 mother–child pairs. The NHBCS cohort obtains drinking water from private well water, which often contains naturally occurring fluoride. In New Hampshire, the median fluoride concentration is 0.4 mg/L, and 9% of water samples exceed the maximum secondary contaminant level of 2.0 mg/L. The NHBCS provides a unique opportunity to examine the dose–response relationships between fluoride and developmental neurotoxicity, while also considering important individual-level variables such as age, various fluoride biomarkers, water consumption habits, fluoride intake from other sources, and timing of exposure.

Ongoing work is examining the impact of prenatal fluoride exposure (measured in urine, water, and toenails) on motor skills, behavior, and IQ. We are also examining the impact of fluoride exposure in infancy from fluoride supplements and formula reconstituted with tap water on neurodevelopmental outcomes.

## Maternal and Developmental Risks from Environmental and Social Stressors (MADRES), Los Angeles, California, USA

### Presented by Carrie Breton and Tracy Bastain

The MADRES study is a prospective pregnancy cohort involving mainly low-income, Hispanic mother–child pairs in urban Los Angeles. Over 60% of MADRES participants live in one of the 10% most environmentally burdened California communities. Additionally, most participants live in historically redlined neighborhoods, which were systematically denied access to economic opportunities [80–82]. MADRES examines environmental and social determinants impacting maternal and child health outcomes both during and after pregnancy. A total of 1,065 participants enrolled in the MADRES cohort during pregnancy from November 2015 through May 2023. Beginning in 2015, the MADRES cohort enrolled pregnant individuals before 30 weeks’ gestation (~ 75% prior to 20 weeks’ gestation) at multiple partner community health prenatal clinics dedicated to medically underserved populations. The protocol, study design and detailed study procedures for the MADRES cohort are described elsewhere [83]. Briefly, data are collected by bilingual and bicultural study staff hired from the target communities, who administer questionnaires orally, either by phone or during in-person study visits. Biospecimen collection, anthropometric measures, and other study procedures for both mothers and children are conducted in person at the MADRES study clinic at the University of Southern California.

Health outcomes data for mothers include hypertensive disorders of pregnancy (HDP), gestational diabetes (GDM), gestational weight gain/retention, cardiometabolic health after pregnancy, depression (antepartum, postpartum and long-term), and allostatic load. Outcomes for children include fetal development measured by ultrasound, birth outcomes (e.g., birthweight, pre-term birth), growth trajectories/obesity, body composition (using EchoMRI™), respiratory health, asthma incidence, and neurodevelopment (including psychomotor development/developmental milestones, infant temperament, neurobehavioral development and autism spectrum disorder traits). Environmental exposure data were assigned to residential addresses of participants across time (e.g., ambient and traffic-related air pollution) or measured in stored biospecimens (e.g., fluoride, per- and polyfluoroalkyl substances (PFAS), metals, emerging chemicals of concern).

We measured maternal urinary fluoride levels, adjusted for urinary specific gravity (MUFsg), in 293 spot urine samples collected from MADRES participants during in-person study visits in the first trimester and 490 samples from the third trimester. Median third trimester concentrations (0.80 mg/L, IQR = 0.59 mg/L) were higher than median first trimester concentrations (0.65 mg/L, IQR = 0.50 mg/L). Participants who were older, had higher income levels, and were non-Hispanic White had higher MUFsg levels. Higher MUFsg concentrations were associated with lower blood mercury levels in the first trimester and were associated with higher blood lead concentrations in the third trimester. We also found that higher MUFsg was associated with higher urinary metals including antimony, barium, cadmium, cobalt, copper, lead, nickel, tin, and zinc in one or both trimesters [49].

The MADRES cohort is also the first US based pregnancy cohort to examine the role of fluoride exposure during pregnancy on neurobehavioral outcomes in children. We examined 229 mother–child pairs who had MUFsg concentrations and had completed the 3-year follow-up visit. Mothers who had reported prenatal smoking were excluded from the analysis. Neurobehavioral outcomes were assessed using the Child Behavior Checklist 1.5–5 (CBCL1.5–5). We examined composite T scores for Total Problems, Internalizing Problems and Externalizing Problems. In this subset, median MUFsg concentration was 0.76 mg/L (IQR = 0.51–1.19). Across an IQR of exposure to MUFsg, we found nearly two-fold increased odds of the Total Problems T score being in the borderline clinical or clinical range (OR = 1.83; 95% CI, 1.17–2.86). We also found that an IQR increase in MUFsg was associated with a 2.29 (95% CI: 0.47, 4.11) point increase in Internalizing Problems composite T scores and a 2.14 (95% CI: 0.29,3.98) point increase in Total Problems composite T score. We further found several significant associations between higher MUFsg levels and higher scores on several syndrome and/or DSM-5 oriented scales including the Emotionally Reactive, Somatic Complaints, Anxiety Problems, and Autism Spectrum Problems scales [63]. Overall, the findings from the MADRES cohort align with previous studies indicating that prenatal exposure to fluoride is linked to negative neurodevelopmental outcomes in children.

## The Infancia y Medio Ambiente (INMA)-Gipuzkoa Cohort, Basque Country, Spain

### Presented by Jesus Ibarluzea-Maurolagoitia and Mikel Subiza-Perez

The INMA birth cohort study is a multicenter research initiative in Spain aimed at analyzing the relationship between prenatal and early postnatal environmental exposures and various developmental and health outcomes. Due to the international interest in the potential neurodevelopmental effects of fluoride exposure, we have conducted studies using the INMA-Gipuzkoa cohort. This cohort is unique because it was the only one established in an area where community water fluoridation was implemented for municipalities with populations exceeding 30,000 during the initial recruitment period from 2006 to 2008, as well as during most follow-ups at ages 2, 4, 6, 8, 11, and 14 years. This setup naturally divided the INMA-Gipuzkoa participants into two groups: those living in municipalities with fluoridated community drinking water and those without. We assessed exposure to fluoride by analyzing urine samples from both mothers (prenatal) and children (postnatal).

In a series of published works [84, 85], we examined the associations between fluoride exposure, cognitive functions, and ADHD. In the first study, we found a positive relationship between fluoride levels in maternal urine and IQ scores in 4-year-old boys; however, this effect could not be confirmed in female participants. In the second study, we did not identify any statistically significant association between prenatal fluoride exposure and ADHD scores at the 8-year follow-up. Nevertheless, we did observe an inverse association between prenatal fluoride exposure and ADHD scores at age 11. Our study results are inconsistent with much of the existing literature, although other studies describe relationships that differ from the expected detrimental effects on neurodevelopment and cognition [56, 86].

We are expanding our analyses to include other relevant health outcomes, such as mental health and behavioral problems [87]. Thus far, we have found no link between prenatal fluoride exposure and internalizing or externalizing problems in data from the follow-ups at ages 8 and 11 (currently under review). In line with Hall (2024) [77], we will investigate potential endocrine disruption effects on thyroid hormones during pregnancy. Finally, using a longitudinal approach, we will examine other cognitive outcomes, such as working memory and hot executive functions.

## The Cincinnati Childhood Allergy and Air Pollution Study (CCAAPS), Cincinnati, Ohio, USA

### Presented by Kelly Brunst and Mauro Martinez

The CCAAPS is part of a larger combined cohort that includes participants from the Health Outcomes & Measures of the Environment (HOME) Study. The CCAAPS and HOME Studies were harmonized to form the Cincinnati Combined Childhood Cohort (C4). Previous descriptions of each cohort’s eligibility, enrollment, and relevant methods are available [88–91]. In 2022, the CCAAPS cohort began conducting studies to examine the impact of childhood urinary fluoride (CUF) concentrations on neurodevelopment outcomes at age 12. We observed higher CUF concentrations to be significantly associated with increased internalizing symptoms including anxiety, depression, and somatization. Furthermore, the effect appeared to be sex-specific with males exhibiting more internalizing symptoms compared to females [87]. The C4 cohort has expanded their work to include studies of potential mechanisms. They conducted an epigenome-wide association study exploring how fluoride exposure impacts the epigenome, specifically DNA methylation, at age 12. Several loci were found to be differentially methylated in response to childhood urinary fluoride concentrations suggesting that these changes may impact the expression of genes involved in neurodevelopment in a sex-specific manner [92].

A limitation of previous studies on the relationship between fluoride exposure and health outcomes is the reliance on biomarkers that only capture narrow windows of exposure. To address this issue, the C4 cohort will implement a discovery and replication study design in collaboration with the PROGRESS cohort. This study will use the tooth matrix as an innovative biomarker, taking advantage of the unique properties of a tooth’s mineralized tissue. This approach would allow us to reconstruct fluoride exposure throughout various developmental stages, starting in utero during the second to third trimesters and continuing until the tooth is shed in childhood. The tooth biomarker has already been suggested as a tool to determine the timing of fluoride exposure during early life and to estimate children’s cumulative, long-term exposure in children [93].

## Illinois Kids Development Study (IKIDS), Urbana-Champaign, Illinois, USA

### Presented by Andrea Aguiar and Susan Schantz

IKIDS is a prospective pregnancy and birth cohort study that was originally designed to assess the cumulative impact of prenatal exposure to endocrine disrupting chemicals (e.g., phthalates, phenols, per- and polyfluoroalkyl substances) as well as maternal prenatal stress on neurodevelopment during infancy and early childhood. In phase 1 of IKIDS, women were recruited at two obstetric clinics in Urbana-Champaign, Illinois–an area where municipal water is fluoridated–and enrolled during the first trimester of pregnancy. Health, diet, lifestyle data, and urine specimens (first morning voids) were collected at five time points during pregnancy. Children were followed prospectively from birth and had cognitive and behavioral assessments conducted at 4–5 months, 7–8 months, and at 2, 3 and 4 years of age. Descriptions of eligibility criteria, enrollment and relevant methods are included in several recent publications [94–97]. Briefly, eligible women were between 18 and 40 years old and willing to provide a fasting blood sample as well as multiple urine samples during pregnancy. They needed to live within 30 min of the IKIDS lab and be able to bring their infants to four appointments at the University of Illinois during the first year of the child's life. Women carrying multiples and women with high-risk pregnancies were ineligible. A total of 535 infants born to women recruited from December 2013 to March 2020 were enrolled in IKIDS at birth. Infrared eye-tracking was used to collect data on physical reasoning, attention, information processing speed and visual recognition memory during infancy. At 2, 3 and 4 years of age, data on early language development and behavior were collected via maternal report using the McArthur Bates Communicative Development Index, the Speech and Language Assessment Scale and the Child Behavior Check List (CBCL).

Through a collaboration with the Human Health Exposure Analysis Resource (HHEAR) the IKIDS cohort is now being leveraged to assess associations between fluoride concentrations in maternal urine samples collected in each trimester of pregnancy and early cognitive and behavioral development of their children. Urine biospecimens from 338 women who had a sample available from each trimester of pregnancy and cognitive/behavioral data available for their child are being analyzed by the HHEAR Lab. Results should be available in 2025 and will add to the still small body of data available on US pregnancy cohorts exposed to what are considered to be optimal levels of municipal water fluoridation.

## Working groups: current challenges and research agenda

### Harmonizing existing cohort data to understand fluoride exposure and its effect on neurodevelopmental outcomes

#### Group members: Susan Schantz, Andrea Aguiar, Marcela Tamayo-Ortiz, Tracy Bastain, and Deborah Watkins

Harmonization of existing cohort data and designing new cohort studies is crucial to understanding the relationship between fluoride exposure and neurodevelopmental outcomes. Similar research conducted across multiple and diverse populations would enable more accurate and reliable conclusions in comprehensive analyses of the overall impact of fluoride exposure on neurodevelopmental health outcomes.

For example, a recent NTP Monograph on fluoride exposure and neurodevelopment [5] reported low confidence in the body of evidence from studies that evaluated fluoride exposure and non-intelligence related cognitive and neurodevelopmental outcomes in children. The authors explained that the low confidence was due to the limited number of studies available, too much heterogeneity in the outcomes measured, ages assessed, and methods used to compare studies of any one outcome directly.

Authors also reported that more prospective cohort studies with individual level exposure measures would help better evaluate associations between lower estimated fluoride exposure (e.g., < 1.5 mg/L fluoride in drinking water) and neurodevelopmental and cognitive outcomes. Existing cohort studies with unanalyzed urinary or drinking water samples and relevant neurodevelopmental outcomes could contribute important data.

## Analytical Challenges and Fluoride Exposure Assessment

### Group members: E. Angeles Martinez-Mier, Gina A. Castiblanco-Rubio, Frank Lippert, Guillermo Tamayo-Cabeza, Alejandra Cantoral, and Mauro Martinez

Four significant challenges exist in assessing fluoride exposure: the technical limitations of existing potentiometric methods, limitations of dietary assessment approaches, and outdated data concerning sources and contributors to fluoride exposure.

- Technical limitations of the current potentiometric methods: The method developed by Taves [42] has proven useful for the recovery and measurement of fluoride in biological and non-biological samples. However, the successful implementation of Taves’ technique largely depends on the technician's skill, it is time-consuming, and it has a steep learning curve when applied in different settings. Analytical methods that are accurate, precise, have lower operator dependency, and are efficient for analyzing larger sample numbers are needed.
- Limitations of current dietary assessment methods: Dietary instruments like FFQs [30] were not designed or validated to consider fluoride intake. As a result, key food items or questions are missing that would be helpful for accurately assessing dietary fluoride intake. Therefore, the group agreed that it is necessary to adapt and validate dietary assessment methods to better capture dietary fluoride intake.
- The use of spot urinary fluoride levels as biomarkers of prenatal fluoride exposure: The discussion highlighted several limitations, including the lack of consensus on the optimal method for adjusting urinary dilution [98], the uncertainties introduced by the physiological changes of pregnancy [99], and the lack of standardized collection protocols. It was agreed that compared to urinary excretion, maternal plasma is subject to lower variability over the course of pregnancy. The adjustment of maternal plasma fluoride levels using maternal hematocrit [100] to account for the increase in plasma volume experienced over the course of pregnancy was also discussed as a possible better alternative to spot urinary fluoride levels.
- Outdated information on sources and contributors of fluoride exposure: The studies that informed current recommendations for dietary fluoride intake were conducted in the late twentieth century [101], before the widespread availability and distribution of processed foods, and prior to the global adoption of fluoride in oral healthcare products. It is imperative to update estimations and identify the contribution of different sources to total fluoride exposure in different population subgroups.
- The need for funding from a variety of sources is required to develop studies that accurately measure fluoride exposure and to develop new analytical techniques capable of handling sample quantities from epidemiological studies.

## Economic impact of fluoride’s effects

### Group members: Marta M. Tellez-Rojo, Jose L. Figueroa, and Elizabeth Roberts

Fluoride exposure, particularly in early childhood, has been linked to neurodevelopmental effects, raising concerns about its potential long-term economic impacts. In areas where fluoride naturally occurs in water and soil, studies have observed adverse outcomes such as reduced intelligence and behavioral problems in children [102–104]. Recent prospective cohort studies involving pregnant women in regions where fluoride is added to tap water or salt for dental health have also raised concerns about developmental neurotoxicity, although the findings are sometimes conflicting [55, 57, 61, 84]. While the neurodevelopmental impact of fluoride exposure is increasingly supported by evidence, its long-term economic consequences remain underexplored.

During the symposium, the group discussed the need to expand research and explore more broadly the hypothesized link between fluoride exposure and long-term economic outcomes. This hypothesis is grounded in the critical role that neurodevelopment plays in shaping cognitive abilities, which directly influences education and future earnings. For instance, impaired cognitive development during key growth phases can lead to delays in school readiness, reduced academic achievement, and lower educational attainment. These educational challenges can then translate into reduced labor market productivity, lower wages, and fewer employment opportunities. Education is a well-established determinant of income, as higher levels of schooling typically result in better job prospects, higher wages, and improved long-term economic stability. Additionally, education is associated with other important life outcomes, such as improved health, longer life expectancy, and reduced likelihood of criminal activity [105–108].

To evaluate these hypothesized links, the group proposed collecting longitudinal data that tracks fluoride exposure and its impacts over time. This would include gathering biomarkers of fluoride exposure (e.g., from urine or plasma samples), measuring neurodevelopment at various stages (from early childhood to adolescence), and monitoring educational outcomes such as academic performance, grade retention, and dropout rates. Additionally, tracking economic outcomes like employment status, income levels, and job stability in adulthood was proposed to assess the broader economic effects of fluoride exposure. The group emphasized collecting data on socioeconomic factors, parental education, and resource access to control for confounding influences. By following individuals over time and across diverse populations, particularly in low- and middle-income countries where data is scarce, we can better understand how fluoride’s neurodevelopmental impacts translate into long-term economic disparities. This research is vital to addressing fluoride's potential role in perpetuating poverty cycles and improving both individual and societal well-being. Considerable funding will be needed to conduct this research which should be made available from a wide range of funding bodies.

## Public’s Knowledge regarding Fluoride

### Group members: Drs. Mikel Subiza-Perez, Jesus Ibarluzea-Maurolagoitia, Christine Till, Carly Goodman, Kelly Brunst, Kyla Taylor, Carrie Breton, Morteza Bashash, and Bruce Lanphear

Research on fluoride toxicity does not occur in a social or political vacuum. In recent years, particularly in the United States, community water fluoridation programs (CWFP) have ignited a contentious debate, leading to significant social opposition and attacks on scientists. On September 24, 2024, Judge Edward Chen ruled that fluoride poses a hazard and mandated that the United States Environmental Protection Agency (EPA) regulate it under the Toxic Substances Control Act.

Previous studies have documented perceptions the risks and benefits of fluoride and examined how these perceptions relate to public support for CWFP in Australia [109], Japan [110], Canada [111], and the United States [112]. However, these studies primarily focused on fluoride’s preventive effects on oral health and did not evaluate potential risks, such as dental fluorosis, bone disease, thyroid, and neurotoxic effects, which are already part of opposition narratives [113].

The growing evidence about fluoride neurotoxicity demands a new survey to examine public perceptions about CWFP. In this context, methods beyond the standard quantitative could help to elucidate whether different sociodemographic groups hold qualitatively different perspectives on these matters [114]. Moreover, a new survey could help us understand how opinions may be shaped by perceptions of risks and benefits of CWFP.

This discussion prompted a reflection on risk communication and the specific role of scientists in this context. Generally, the goals of health-related risk communication are to align the perceived risk with the actual risk associated with a certain exposure for the community [115] and facilitate protective behaviors if they are needed. The relationship between fluoride and human health is complex and involves several contrasting factors. These include the need to prevent dental caries while also considering potential adverse effects, such as thyroid disruption and developmental neurotoxicity. Additionally, the effectiveness and implications of local exposures (like those from toothpaste) versus systemic exposures (such as those from drinking water) are actively debated. The timing of exposure during developmental periods and the thresholds for dose–response also add to the complexity. All these factors contribute to the uncertainty surrounding the objectives and content of fluoride-related communications, particularly in the absence of a consensus on the risks and benefits of fluoride at levels used for drinking water treatment.

What topics should we communicate regarding fluoride? How should we convey this information, and who is our target audience? What specific health outcomes do we aim to enhance understanding of? Additionally, what ethical considerations should guide our communication about fluoride? These questions are still relevant today and deserve further consideration before establishing communication agendas and strategies related to fluoride.

## Key terminology

The participants also felt the need to summarize the terminology used in research on the effects of fluoride on human health (Table 1).

**Table 1:** Terminology used in research on the effects of fluoride on human health

Key terms	Specifics
Timing of exposure	pre-eruptive, post-eruptive
Timing of measuring fluoride exposure	contemporary, historical
Mode of administration or source of fluoride	systemic exposure or intake, topical exposure or intake
Methods for measuring exposure	dietary intake (e.g., determined through food-frequency questionnaires, 24-h dietary recall, or duplicate plate methods), biomarkers (e.g., urine, toenails, blood)
Types of fluoride	organic (e.g., PFAS), inorganic (e.g., sodium fluoride, stannous fluoride)

## Conclusions and research agenda

The symposium highlighted the need for ongoing research, collaboration, and effective communication to address the complexities of fluoride exposure and its effects on human health. Conflicting findings in studies on fluoride and neurodevelopment across various populations do not invalidate each other's results; instead, they raise questions about the missing pieces of the puzzle and the need for continued collaboration and effective communication among scientists to harmonize study designs and data, with the goal of preserving fluoride’s benefits while minimizing any potential harm to human health. The collaboration of experts identified the following research needs during presentations and group discussions:

- Adaptation/customization of dietary assessment tools customized for fluoride exposure assessment and collection of updated information on contemporary sources of exposure
- Development of high throughput analytical methods for the measurement of fluoride in biological samples
- Selection of biomarkers that consider the metabolism of fluoride and cover the susceptibility windows of the health outcome of interest
- Adjustment of study designs of existing and still recruiting as well as future birth cohort studies on environmental toxins to assess fluoride exposure and neurodevelopment, as none of the existing birth cohort studies were designed to specifically study fluoride exposure (e.g., selection of biomarkers, collection intervals, diet exposure assessment)
- Conduct of broader environmental health cohort studies that incorporate high-quality biomonitoring of waterborne neurotoxicants (such as fluoride, arsenic, lead, mercury), repeated measures of exposure, and inclusion of key covariates (e.g., socio-economic status, diet, iodine) using methods for the statistical analysis of environmental mixtures
- Research addressing low-level fluoride exposures that are currently considered safe
- Establishment of new and continuation of existing partnerships, particularly international or multidisciplinary ones, to reinforce the need for broader collaborations, beyond scientific harmonization
- Need for funding from government funding bodies, foundations, and industry globally on above mentioned future research, tool development, and methodological improvements
- Harmonization of existing cohort data to conduct similar research across diverse populations
- Tailoring effective communication strategies to convey the complexities of fluoride exposure and its health impacts to the public
- More effective scientific communication to avoid misrepresentation of study findings
- Exploration of the potential link between fluoride exposure and long-term economic impacts

## Data Availability

Not applicable.

## Abbreviations

ADHD: Attention-deficit/hyperactivity disorder
C4: Cincinnati Combined Childhood Cohort
CBCL: Child Behavior Check List
CBCL1.5–5: Child Behavior Checklist 1.5–5
CCAAPS: Cincinnati Childhood Allergy and Air Pollution Study
CI: Confidence interval
CUF: Childhood urinary fluoride
CWFP: Community water fluoridation programs
d: Day(s)
DNA: Deoxyribonucleic acid
DSM-5: Diagnostic and Statistical Manual of Mental Illnesses
e.g.: Exempli gratia (Latin); for example (English)
EchoMRI™: Magnetic resonance imaging by Echo
ELEMENT: Early Life Exposures in Mexico to ENvironmental Toxicants study
EPA: Environmental Protection Agency
et al.: Et alii (Latin); and others (English)
FFQs: Food frequency questionnaires
GDM: Gestational diabetes
h: Hour(s)
HbA1c: Hemoglobin A1C
HDP: Hypertensive disorders of pregnancy
HHEAR: Human Health Exposure Analysis Resource
HMDS: Hexamethyldisiloxane
HOME: Health Outcomes & Measures of the Environment
IKIDS: Illinois Kids Development Study
INMA: Infancia y Medio Ambiente
IQ: Intelligence quotient
IQR: Interquartile range
MADRES: Maternal and Developmental Risks from Environmental and Social Stressors
mg F/kg/d: Milligram of fluoride per kilogram and day
mg/L: Milligram per liter
MIREC: Maternal-Infant Research on Environmental Chemicals study
MUFsg: Maternal urinary fluoride levels, adjusted for urinary specific gravity
NHBCS: New Hampshire Birth Cohort Study
NTP: National Toxicology Program
OR: Odds ratio
PFAS: Per- and polyfluoroalkyl substances
PROGRESS: Programming Research in Obesity, GRowth, Environment, and Social Stress study
WHO: World Health Organization
μg/g: Microgram per gram
